# Expression and regulation of avian beta-defensin 8 protein in immune tissues and cell lines of chickens

**DOI:** 10.5713/ajas.17.0836

**Published:** 2018-03-13

**Authors:** Deivendran Rengaraj, Anh Duc Truong, Hyun S. Lillehoj, Jae Yong Han, Yeong Ho Hong

**Affiliations:** 1 Department of Animal Science and Technology, Chung-Ang University, Anseong 17546, Korea; 2 Department of Agricultural Biotechnology and Research Institute of Agriculture and Life Sciences, College of Agriculture and Life Sciences, Seoul National University, Seoul 08826, Korea; 3 Department of Biochemistry and Immunology, National Institute of Veterinary Research, 86 Truong Chinh, Dong Da, Hanoi 100000, Vietnam; 4 Animal Biosciences and Biotechnology Laboratory, Agricultural Research Services, United States Department of Agriculture, Beltsville, MD 20705, USA

**Keywords:** Chicken, Avian beta-defensin 8, Immune Tissues, Immune Cells, Protein Expression

## Abstract

**Objective:**

Defensins are a large family of antimicrobial peptides and components of the innate immune system that invoke an immediate immune response against harmful pathogens. Defensins are classified into alpha-, beta-, and theta-defensins. Avian species only possess beta-defensins (AvBDs), and approximately 14 AvBDs (AvBD1–AvBD14) have been identified in chickens to date. Although substantial information is available on the conservation and phylogenetics, limited information is available on the expression and regulation of AvBD8 in chicken immune tissues and cells.

**Methods:**

We examined AvBD8 protein expression in immune tissues of White Leghorn chickens (WL) by immunohistochemistry and quantitative reverse transcription-polymerase chain reaction (RT-qPCR). In addition, we examined AvBD8 expression in chicken T-, B-, macrophage-, and fibroblast-cell lines and its regulation in these cells after lipopolysaccharide (LPS) treatment by immunocytochemistry and RT-qPCR.

**Results:**

Our results showed that chicken AvBD8 protein was strongly expressed in the WL intestine and in macrophages. *AvBD8* gene expression was highly upregulated in macrophages treated with different LPS concentrations compared with that in T- and B-cell lines in a time-independent manner. Moreover, chicken AvBD8 strongly interacted with other AvBDs and with other antimicrobial peptides as determined by bioinformatics.

**Conclusion:**

Our study provides the expression and regulation of chicken AvBD8 protein in immune tissues and cells, which play crucial role in the innate immunity.

## INTRODUCTION

The immune system comprises the innate and adaptive immune systems. The innate immune system includes immune cells, antimicrobial peptides, enzymes, and proinflammatory factors that induce immediate immune response against harmful pathogens [1]. Of these components of the innate immune system, antimicrobial peptides appear to be very active in eliminating microbes such as bacteria [2]. Defensins are a large family of antimicrobial peptides found in vertebrates and are classified into three major categories, namely, alpha-, beta-, and theta-defensins. Alpha-defensins are exclusively found in mammals, beta-defensins are found in most vertebrates, and theta-defensins are exclusively found in primates [3,4]. Defensins are structurally distinct cationic and cysteine-rich peptides and are classified according to the size and spacing pattern of six cysteine motifs [4]. Vertebrate beta-defensin genes have evolved from a single beta-defensin-like gene, alpha-defensin genes have evolved from beta-defensin genes, and theta-defensin genes have evolved from alpha-defensin genes [5]. Defensins play a crucial role in inducing innate immunity and protect hosts from various pathogens, including bacteria, fungi, protozoa, and some enveloped viruses [5,6].

Birds only possess beta-defensins. Several studies performed over the last decade have identified, profiled the expression of, and analyzed the functions of avian beta-defensins (AvBDs). Approximately 14 AvBDs (AvBD1–AvBD14) have been identified in the chicken to date; moreover, several AvBDs have been identified in other avian species such as the turkey, ostrich, king penguin, mallard duck, and king pigeon [7]. Several studies have reported the expression and crucial role of chicken AvBDs in inducing innate immunity against various pathogens [1,2,6,8,9]. However, little is known about the expression and regulation of AvBD8 protein in immune tissues and cells. In this study, we examined AvBD8 protein expression in the thymus, spleen, liver, intestine, and ceca of White Leghorn (WL) chickens by immunohistochemistry and quantitative reverse transcription-polymerase chain reaction (RT-qPCR). Moreover, we examined AvBD8 expression in chicken T-, B-, macrophage-, and fibroblast-cell lines by immunocytochemistry and its regulation in these cells after treatment with lipopolysaccharide (LPS, which was obtained from *Salmonella enterica* (*S. enterica*)serotype Typhimurium) by RT-qPCR.

## MATERIALS AND METHODS

### Experimental birds, care, and sample collection

Healthy WL chickens were kindly provided by the Animal Genetic Engineering Laboratory of the Department of Agricultural Biotechnology, Seoul National University, Korea. Care and experimental use of these chickens were approved by the Institute of Laboratory Animal Resources, Seoul National University, Korea. The thymus, spleen, liver, small intestine (jejunum region), and ceca were collected from three independent male WL chickens aged 25 weeks, were frozen in liquid nitrogen, and were stored at −85°C. Chicken CU91 T-, DT40 B-, HD11 macrophage-, and OU2 fibroblast-cell lines (kindly provided by the Avian Disease and Oncology Laboratory of the USDA-ARS) were cultured in Dulbecco’s modified Eagle’s medium (Invitrogen, Carlsbad, CA, USA) containing 100 IU/mL penicillin (Sigma-Aldrich, St. Louis, MO, USA), 100 mg/mL streptomycin (Sigma-Aldrich, USA), and 10% heat-inactivated fetal bovine serum (Invitrogen, USA) in a humidified 5% CO_2_ atmosphere at 41°C [10].

### Analysis of AvBD8 expression by immunohistochemistry, RT-qPCR, and immunocytochemistry

Immunohistochemical analysis was performed to examine AvBD8 expression in the thymus, spleen, liver, small intestine, and ceca of male WL chickens in triplicate with three independent samples. Briefly, the frozen sections (thickness, 10 μm) of the tissues were fixed in 4% paraformaldehyde (PFA) for 1 h at room temperature [11]. Next, the sections were washed in 1× phosphate-buffered saline (PBS) and were incubated in 1% Triton X-100 for 10 min at room temperature. Next, the sections were blocked with 1% bovine serum albumin (BSA) in 1× PBS and were incubated overnight with polyclonal rabbit anti-AvBD8 immunoglobulin G (IgG) antibody (dilution, 1:200) (Produced by AbFrontier, Seoul, Korea) in 1% BSA at 4°C. Next, the sections were incubated with Alexa Fluor 488-conjugated goat anti-rabbit IgG secondary antibody (dilution, 1:200; Thermo Fisher Scientific, Waltham, MA, USA) in 1% BSA for 1 h. Control sections received secondary antibody only. Finally, the sections were mounted with Vectashield mounting medium for performing fluorescence analysis with 4′,6-diamidino-2-phenylindole (DAPI; Vector Laboratories, Burlingame, CA, USA) and were photographed using a fluorescence microscope (Nikon Corporation, Tokyo, Japan).

RT-qPCR was performed to examine the expression of *AvBD8* in the thymus, spleen, liver, small intestine, and ceca of male WL chickens in triplicate with three independent samples. First, total RNA was extracted from the tissues by using TRIzol reagent (Invitrogen, USA). Next, 2 μg total RNA was reverse transcribed to cDNA by using CellScript 5× all-in-one 1st-Strand cDNA Synthesis Master Mix (Cellsafe, Yongin, Korea), according to manufacturer’s protocols. The qPCR was performed using Light Cycler 96 real-time PCR System (Roche Diagnostics, Indianapolis, IN, USA) in a 20-μL reaction mixture containing 1 μL cDNA, 10 μL 2× FastStart Universal SYBR Green Master Mix (Roche Diagnostics, USA), and 10 pmol each of forward (5′-TCC TCA CTG TGC TCC AAA GC-3′) and reverse (5′-GTG CCC AAA GGC TCT GGT AT-3′) primers of *AvBD8* (NM_001001781). The primers were designed using the NCBI primer-BLAST tool and were synthesized by Genotech Co. Ltd. (Daejeon, Korea). Thermal conditions for performing qPCR are as follows: initial incubation at 95°C for 5 min; 40 cycles of denaturation at 95°C for 30 s, annealing at 60°C for 30 s, and extension at 72°C for 30 s; and termination by final incubation at dissociation temperatures 95°C (10 s), 65°C (60 s), 97°C (1 s), and 37°C (30 s). *AvBD8* expression was quantified after normalization with the chicken glyceraldehyde-3-phosphate dehydrogenase gene (*GAPDH*; NM_204305). *GAPDH* was amplified using primers 5′-TGC TGC CCA GAA CAT CAT CC-3′ (forward primer) and 5′-ACG GCA GGT CAG GTC AAC AA-3′ (reverse primer).

Immunocytochemical analysis was performed to examine AvBD8 expression in chicken T-, B-, macrophage-, and fibroblast-cell lines. Briefly, the cells were cultured on a chamber slide (Thermo Fisher Scientific, USA) and were fixed in 4% PFA for 15 min at room temperature. After washing with 1× PBS, the cells were incubated with 0.25% Triton X-100 for 10 min at room temperature. After blocking, the cells were incubated overnight with the rabbit anti-AvBD8 primary antibody (dilution, 1:100) at 4°C, followed by incubation with the Alexa Fluor 488-conjugated goat anti-rabbit IgG secondary antibody (dilution, 1:500) for 1 h. Control cells received secondary antibody only. Finally, the cells were treated with DAPI, mounted, and imaged using EVOS FLoid Cell Imaging Station (Life Technologies, Carlsbad, CA, USA). Immunocytochemistry was performed in triplicate with three independent samples.

### Analysis of cytotoxicity and *AvBD8* expression in LPS-treated immune cell lines

CU91 T-, DT40 B-, HD11 macrophage-, and OU2 fibroblast-cell lines were cultured at a density of 1×10^6^ cells/well in six-well plates and were treated with medium alone or 1, 3, and 5 μg/mL LPS obtained from *S. enterica* serotype Typhimurium (Sigma-Aldrich, USA) for approximately 12, 24, and 48 h. Simultaneously, approximately 50,000 cells/well were cultured in a 96-well microtiter plate and were treated with the same concentration of LPS (converted to 100 μL) for determining the cytotoxicity of LPS by cell counting. Cell counting was performed using cell counting kit-8 (CCK-8; Dojindo Molecular Technologies, Mashikimachi, Kumamoto, Japan), according to the manufacturer’s protocols. LPS-treated cells cultured in the six-well plates were harvested at 12, 24, and 48 h, and *AvBD8* expression in these cells was analyzed by performing RT-qPCR. Total RNA was extracted from control and LPS-treated cells by using the TRIzol reagent. Next, 2 μg of the total RNA was reverse transcribed to cDNA by using the CellScript 5× all-in-one 1st-Strand cDNA Synthesis Master Mix. All other procedures of qPCR were the same as those mentioned above. The cytotoxicity and *AvBD8* expression in LPS treated immune cell lines were performed in triplicate with three independent samples.

### Protein-protein interaction analysis of AvBD8

Interactions between AvBD8 and other antimicrobial proteins/peptides were determined using the Search Tool for the Retrieval of Interacting Genes/Proteins (STRING) database (version 10.5) [12]. Protein-protein interactions were analyzed under medium confidence (score, 0.400) by using prediction methods such as text mining, experiments, databases, co-expression, co-occurrence, neighborhood, and gene fusion [13]. The number of interactors was set to “no more than 50” for the first shell and “none” for the second shell, and finally an evidence-based network was retrieved.

### Statistical analysis

Data are represented as mean±standard error of mean of three independent experiments for each group (n = 3) and were analyzed using SAS 9.4 statistical program (SAS Institute, Cary, NC, USA). Control and experimental groups were compared using Duncan’s multiple range test. Statistical significance is indicated in the order * p<0.05, ** p<0.01, and *** p<0.001.

## RESULTS AND DISCUSSION

### Expression and regulation of chicken AvBD8 in immune tissues

**Figure 1:**
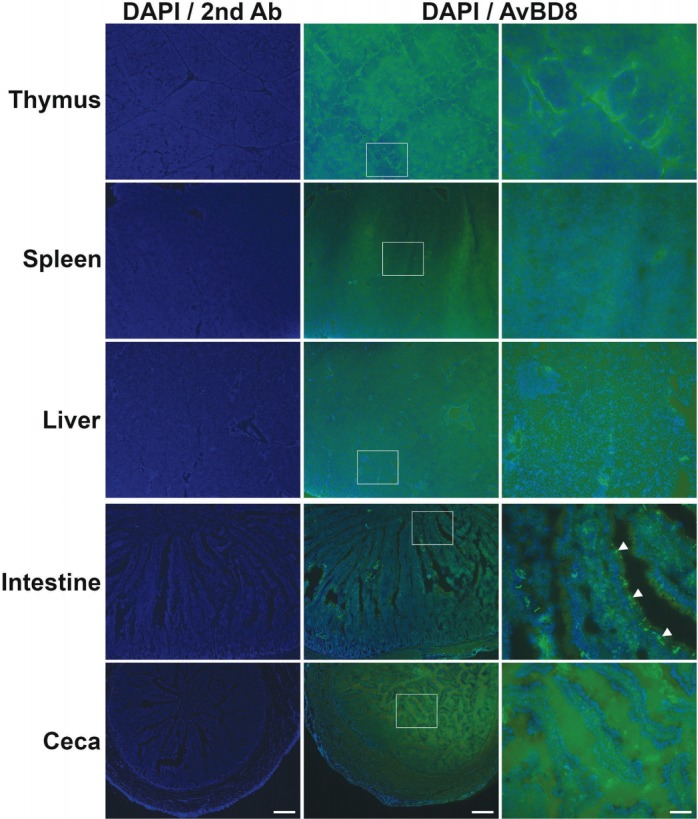
Determination of AvBD8 protein expression in immune tissues of male WL chickens by performing immunohistochemical analysis. Immunohistochemical analysis was performed to assess AvBD8 protein expression in the thymus, spleen, liver, small intestine, and ceca of male WL chickens aged 25 weeks. Frozen sections were incubated with the rabbit anti-AvBD8 primary antibody, followed by incubation with the Alexa Fluor 488-conjugated goat anti-rabbit IgG secondary antibody, and were counterstained with 4′,6-diamidino-2-phenylindole. Control sections were incubated with secondary antibody only. Boxed region in the middle column is enlarged in the right column. AvBD8, avian beta-defensin 8; WL, White Leghorn; IgG, immunoglobulin G. Scale bar: 200 μm (left and middle columns) and 50 μm (right column). Arrowheads indicate strong AvBD8 signal in the intestinal mucosal layer.

**Figure 2:**
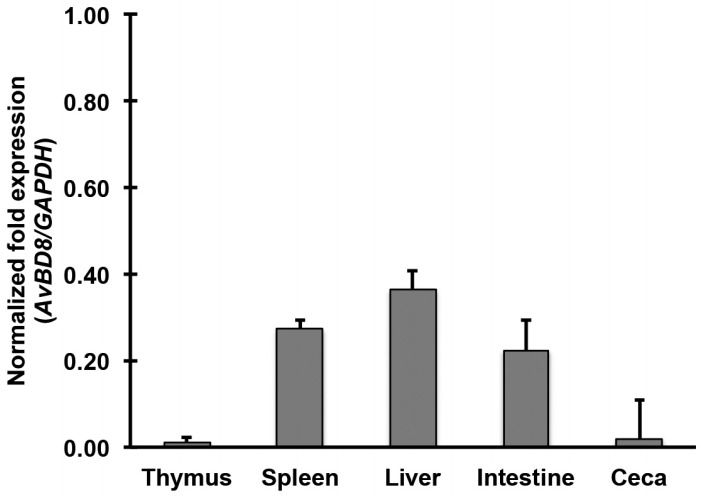
Determination of *AvBD8* expression in the immune tissues of male WL chickens by performing RT-qPCR. RT-qPCR was performed to examine *AvBD8* expression in the thymus, spleen, liver, small intestine, and ceca of male WL chickens aged 25 weeks. cDNAs synthesized using RNAs obtained from the immune tissues were amplified using *AvBD8*-specific primers, and target gene expression was normalized to that of *GAPDH*. *AvBD8*, avian beta-defensin 8; WL, White Leghorn; RT-qPCR, quantitative reverse transcription-polymerase chain reaction; *GAPDH*, glyceraldehyde-3-phosphate dehydrogenase. Data are represented as mean±standard error of mean of three independent experiments (n = 3).

In this section, we examined AvBD8 protein expression in the thymus, spleen, liver, small intestine (jejunum region), and ceca of male WL chickens (age, 25 weeks) by performing immunohistochemical analysis. The male WL chickens showed strong AvBD8 expression in the mucosal layer of the small intestine and medium-to-low AvBD8 expression in other immune tissues (Figure 1). Beta-defensins are important components of the innate immune system in the intestinal mucosal tissue, which is a major site for the entry of several pathogens [14]. To validate this result, we examined *AvBD8* gene expression in the immune tissues obtained from male WL chickens by performing RT-qPCR. In contrast to immunohistochemistry, results of RT-qPCR showed that *AvBD8* expression level was similar in the spleen, liver, and intestine and was low in the thymus and ceca of male WL chickens (Figure 2). This may indicate the sensitivity and accuracy between qualitative and quantitative techniques. Previous studies have reported high, moderate, low, or no *AvBD8* gene expression in tissues of young healthy chickens by performing simple reverse transcription PCR (RT-PCR). Lynn et al [4] reported high *AvBD8* gene expression in the liver, gall bladder, kidney, and testis of 3-week-old healthy male chickens. Moreover, Xiao et al [5] reported high *AvBD8* gene expression only in the liver of 8-week-old healthy chickens. Recently, Hamad et al [15] reported high *AvBD8* gene expression in the thymus, spleen, and bursa of 3- to 4–week-old healthy male turkeys. These studies suggest that *AvBD8* gene/protein expression is high in young birds and decreases in aged birds.

Since AvBD8 is an antimicrobial peptide, there have been several studies on the expression and regulation of AvBD8 in the chicken tissues in response to various microbes. Results of RT-qPCR performed in our previous study [6] showed intestine-specific *AvBD8* gene expression compared with that in the crop and spleen of necrotic enteritis (NE)-infected Ross and Cobb broiler chickens. In another study, NE-infected Ross chickens treated with selenium showed increased *AvBD8* expression in the intestinal mucosa and spleen in a dose-dependent manner [16]. A study by Lee et al [2] showed high *AvBD8* expression in the liver and bone marrow of inbred Leghorn chicken line Ghs-6; however, the antibacterial activity of AvBD8 against *Escherichia coli* (*E. coli*) was not effective. A study by Su et al [1] reported variable regulation of *AvBD8* expression in the intestinal samples of Ross broiler male chickens infected with *Eimeria acervulina*, *Eimeria maxima*, or *Eimeria tenella*. Collectively, this section suggests that AvBD8 expression and regulation in immune tissues is associated with the age and breed of chickens and with specific-pathogens that infect these chickens.

### Expression and regulation of chicken AvBD8 in immune cell lines

**Figure 3:**
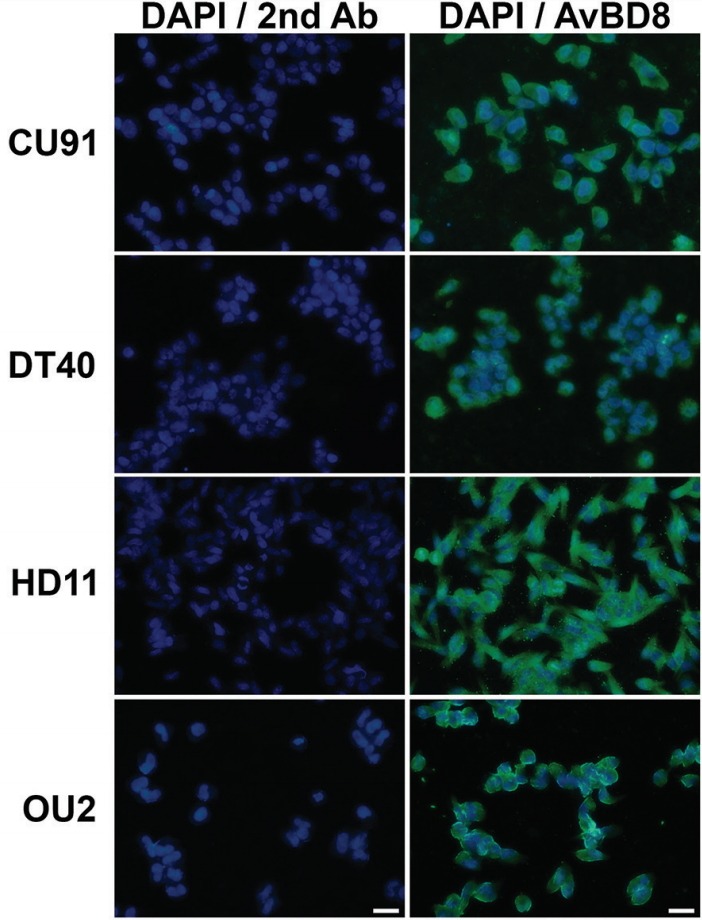
Determination of AvBD8 protein expression in immune cell lines by performing immunocytochemical analysis. Cultured chicken CU91 T-, DT40 B-, HD11 macrophage-, and OU2 fibroblast-cell lines were incubated with the rabbit anti-AvBD8 primary antibody, followed by incubation with the Alexa Fluor 488-conjugated goat anti-rabbit IgG secondary antibody, and were counterstained with 4′,6-diamidino-2-phenylindole. Control cells were incubated with secondary antibody only. AvBD8, avian beta-defensin 8; IgG, immunoglobulin G. Scale bar: 20 μm.

**Figure 4:**
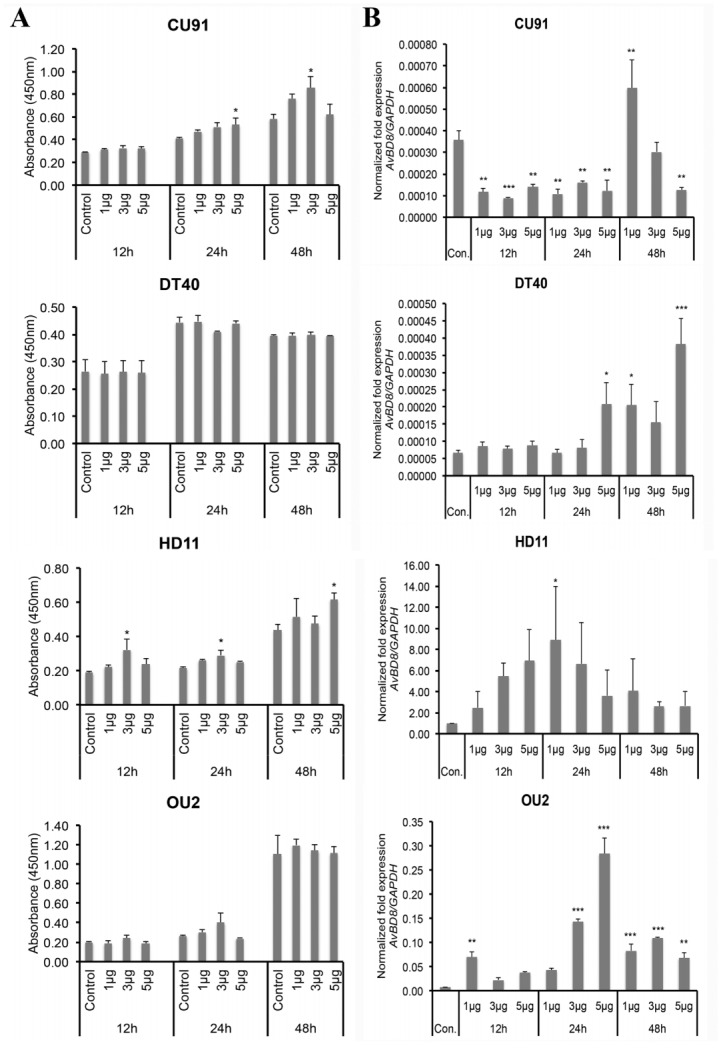
Determination of cytotoxicity and *AvBD8* gene regulation in LPS-treated immune cell lines. (A) CU91 T-, DT40 B-, HD11 macrophage-, and OU2 fibroblast-cell lines were cultured in a 96-well microtiter plate at a density of 50,000 cells/well and were treated with medium alone or 1, 3, and 5 μg/mL (converted to 100 μL) LPS for approximately 12, 24, and 48 h. Next, the LPS-treated and untreated control cells were counted at appropriate time intervals by using the CCK-8 kit. (B) The cell lines were cultured in six-well plates at a density of 1×10^6^ cells/well and were treated with medium alone or 1, 3, and 5 μg/mL LPS for approximately 12, 24, and 48 h. cDNAs synthesized using RNAs obtained from LPS-treated and untreated control cells were amplified using *AvBD8*-specific primers, and target gene expression was normalized to that of *GAPDH*. *AvBD8*, avian beta-defensin 8; LPS, lipopolysaccharide; *GAPDH*, glyceraldehyde-3-phosphate dehydrogenase. Data are represented as mean±standard error of mean of three independent experiments (n = 3). Statistical significance between control and treatment groups: * p<0.05, ** p<0.01, and *** p<0.001.

The expression of chicken AvBD8 protein in CU91 T-, DT40 B-, HD11 macrophage-, and OU2 fibroblast-cell lines was examined by performing immunocytochemical analysis with the anti-AvBD8 primary antibody. Our results showed that AvBD8 expression was higher in macrophages than in T-, B-, and fibroblast-cell lines (Figure 3). Next, we treated T-, B-, macrophage-, and fibroblast-cell lines with medium alone (control) or 1, 3, and 5 μg/mL LPS for approximately 12, 24, and 48 h. Results of the cell counting assay showed that LPS treatment did not affect the proliferation of immune cells and fibroblasts compared with that of respective control cells (Figure 4A). Results of RT-qPCR showed differential regulation of *AvBD8* gene expression in these cells in a dose- and time- dependent/independent manner compared with that in control cells. In T-cell line, *AvBD8* expression was significantly upregulated after treatment with 1 μg/mL LPS for 48 h. In B-cell line, *AvBD8* expression was significantly upregulated after treatment with 5 μg/mL LPS for 24 h and after treatment with 1 to 5 μg/mL LPS for 48 h. In macrophages, *AvBD8* expression was upregulated after treatment with all the concentrations of LPS for 12 to 48 h, however 1 μg/mL LPS for 24 h was significant. In fibroblasts, *AvBD8* expression was significant and high after treatment with 5 μg/mL LPS for 24 h when compared to all other treatments (Figure 4B). In addition, comparison of *AvBD8* gene expression in untreated control cell lines of T, B, macrophage, and fibroblast showed higher *AvBD8* expression in macrophages (Figure 4), which was similar to AvBD8 protein expression determined by immunocytochemical analysis (Figure 3). The above results suggest that the expression of AvBD8 was high in macrophages than that of other immune cells tested, and upregulated in a dose- and time-independent manner in response to LPS treatment. The innate immune response is the immediate response against infectious agents mediated largely by white blood cells such as neutrophils and macrophages. In macrophages, the infectious agent is degraded within the phagosomes, and components of the pathogen are presented to the cells involved in the adaptive immune response [17]. The reason for the lower expression of AvBD8 in non-macrophage cells may be due to their secondary response against infectious agents, however a dose- and time-dependent upregulation of AvBD8 in these cells needs further extensive analysis.

*S. enterica* serotype Typhimurium is a broad-host-range serotype that frequently causes diseases in various species, including humans, livestock, domestic fowl, rodents, and birds [18]. LPS derived from the outer membrane of *S. enterica* serotype Typhimurium induces a strong immune response [19]. Antimicrobial peptides, which are essential components of the innate immune system in plants, flies, avians, mammals, and humans, are suggested to disrupt the membrane integrity of microbes [4]. Specifically, AvBDs play an important role in immune regulation, such as anti-inflammatory role, by blocking LPS-induced inflammation and by promoting wound healing [20]. *AvBD8* expression is significantly altered in peripheral blood leukocytes of Ross broiler chickens infected with *S. typhimurium* and *Campylobacter jejuni* [21]. Besides endogenous AvBDs, synthetic or target-modified AvBD peptides also show antimicrobial activity against various bacteria [2,8,22]. Synthetic chicken AvBD11 is predominantly active against intestinal pathogens *S. typhimurium* and *Listeria monocytogenes* [8]. The antimicrobial activity of chicken AvBD8 against *E. coli*, *L. monocytogenes*, *S. typhimurium*, *S. typhimurium* phoP– mutant, and *Streptococcus pyogenes* can be significantly increased by performing targeted amino acid substitution, which increases the peptide charge of proteins [22]. The expression and upregulation of AvBD8 in immune cell lines, especially macrophages, performed in the present study highlights the crucial role of AvBDs in innate immune response in chickens.

### Interactions of AvBD8 with other antimicrobial proteins/peptides

**Figure 5:**
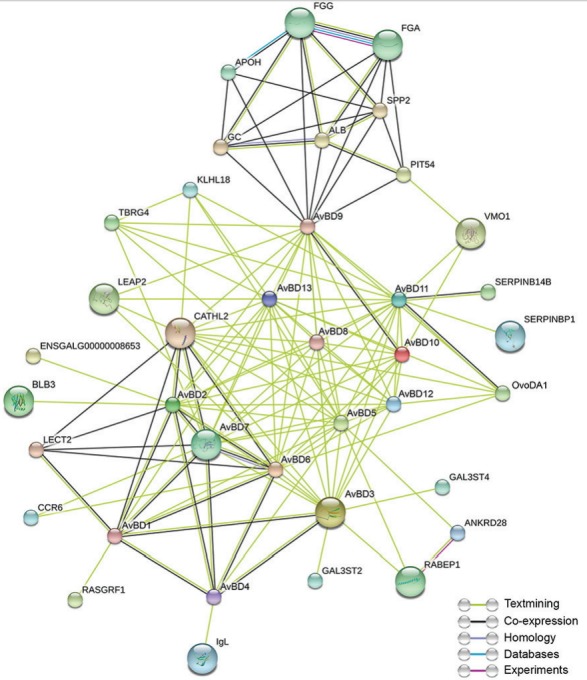
Protein–protein interactions of chicken AvBD8. Evidence-based medium-confidence interactions (score, 0.400) of AvBD8 with other AvBDs and other antimicrobial proteins were identified using the STRING program. Maximum interactors: first shell, no more than 50; second shell, none. ALB, albumin; ANKRD28, ankyrin repeat domain 28; APOH, apolipoprotein H; AvBD1–AvBD14, avian beta-defensin 1–14; BLB3, major histocompatibility complex class II beta chain BLB3; CATHL2, cathelicidin-2; CCR6, C-C motif chemokine receptor 6; GC, a vitamin D-binding protein; ENSGALG00000008653, uncharacterized protein (705 amino acids); FGA, fibrinogen alpha chain; FGG, fibrinogen gamma chain; GAL3ST2, galactose-3-*O*-sulfotransferase 2; GAL3ST4, galactose-3-*O*-sulfotransferase 4; IgL, Ig lambda chain V-1 region; KLHL18, Kelch-like family member 18; LEAP2, liver-enriched antimicrobial peptide 2; LECT2, leukocyte cell-derived chemotaxin 2; OvoDA1, ovodefensin A1; PIT54, PIT54 protein; RABEP1, rabaptin, RAB GTPase-binding effector protein 1; RASGRF1, Ras protein-specific guanine nucleotide-releasing factor 1; SERPINB14B, ovalbumin-related protein Y (SERPINB14B); SERPINBP1, uncharacterized protein (232 amino acids); SPP2, secreted phosphoprotein 2; TBRG4, transforming growth factor-beta regulator 4; VMO1, vitelline membrane outer layer 1 homolog.

At present, 14 chicken AvBDs have been identified, and genes encoding these AvBDs are densely clustered within approximately 86-kb region of chromosome 3 (3q3.5–3q3.7) [7,23]. Several earlier studies have reported that the chicken AvBDs are highly conserved due to the presence of beta-defensin functional domain that spans 32 to 36 amino acids and are evolutionarily related to other vertebrate defensins [4,5,8,23, 24]. Analysis of protein–protein interactions mainly helps in determining co-expressed and co-functional proteins that trigger or inhibit some cellular functions [25]. Interactions of AvBD8 with other AvBD family members and other antimicrobial proteins were examined using the STRING program (Figure 5). The STRING program identified AvBD1–AvBD13 as gallinacin 1–13 (GAL1–GAL13) but could not identify AvBD14. AvBDs were originally referred to as gallinacins; however, their nomenclature was changed to AvBDs to prevent confusion in naming avian defensins [7]. Our results indicated strong interactions among AvBD family proteins, and we identified 25 functional partners of AvBDs. Specifically, chicken leukocyte cell-derived chemotaxin-2 (LECT2) and cathelicidin-2 (CATHL2) showed highest score (0.920 and 0.919, respectively) for interaction with a few AvBDs. Expression of LECT2, CATHL2, AvBD1, and a few other proteins with antibacterial functions is upregulated in chicken macrophages infected with *S. enteritidis* [26]. However, the majority of predicted interactions among AvBDs and between AvBDs and other antimicrobial proteins were merely derived from textmining data and lacks experimental evidence. Therefore, co-expression and co-functional based studies among AvBDs and between AvBDs and other antimicrobial proteins should be conducted to provide experimental evidence.

## CONCLUSION

AvBD8 protein is strongly expressed in the WL chicken intestine compared with that in the thymus, spleen, liver, and ceca and in HD11 macrophage cell line compared with that in CU91 T-, DT40 B-, and OU2 fibroblast-cell lines. Chicken *AvBD8* gene expression is upregulated in HD11 macrophages treated with different concentrations of LPS in a dose- and time-independent manner. Chicken AvBDs strongly interact with each other and with other antimicrobial peptides such as LECT2 and CATHL2. Collectively, our study highlights the expression and regulation of chicken AvBD8 protein in immune tissues and cells, which play crucial role in the innate immunity.
